# Hip joint function and reconstruction of the anterior femoral offset in patients with short stem vs. conventional THA

**DOI:** 10.1038/s41598-023-29513-z

**Published:** 2023-02-10

**Authors:** S. Budde, K. Tonin, E. Jakubowitz, B. Welke, A. Obermeier, C. Hurschler, H. Windhagen, M. Schwarze

**Affiliations:** 1 Department for Orthopaedics, Hannover Medical School, Anna-Von-Borries-Str. 1-7, 30625 Hannover, Germany; 2 Laboratory for Biomechanics and Biomaterials, Hannover Medical School, Anna-Von-Borries-Str. 1-7, 30625 Hannover, Germany

**Keywords:** Musculoskeletal system, Outcomes research

## Abstract

In cases where mobility and joint function are impaired after implantation of a THA, weakening of hip movement in both extension/flexion and adduction/abduction may play a role due to shortening of the physiological lever arm of the hip muscles. Mechanical factors of influence include the lateral femoral offset, which affects the lever arm, and the antetorsion angle of the hip prosthesis, which affects the anterior femoral offset. This study aimed to investigate the effect of an altered antetorsion angle of the implant on the hip moments and gait patterns of the patient. For this study, 13 patients with a conventional stem on one side and a calcar-guided short stem implanted on the contralateral side were included. To determine the maximum hip moment, tests were performed on a dynamometer in extension/flexion and adduction/abduction in addition to gait analysis. As a control, a comparison was made with data from a reference group of 30 healthy subjects. Both implants showed similar symmetry indices. There was a significant difference between the implants for adduction moments (p < 0.001). The ratios between the directions of moments showed no significant differences. The joint function measured by isokinetic measurements and gait analysis remains comparable to the healthy control group after short stem arthroplasty, but shows slight changes after conventional stem arthroplasty.

## Introduction

**Figure 1:**
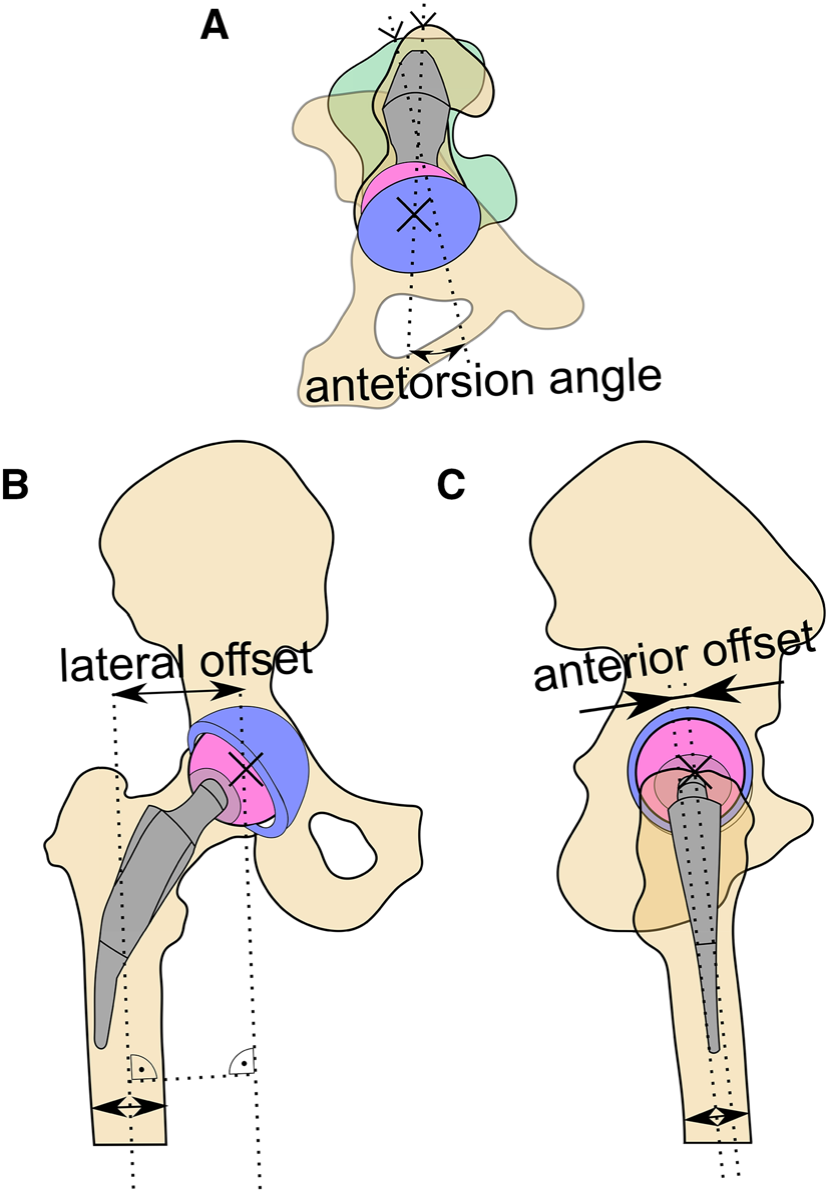
Schematic of the femoral offsets and the antetorsion angle in (**A**) cranial, (**B**) ventral, and (**C**) lateral view. The antetorsion angle is determined as the angle between a line passing through the lateral and medial epicondyle of the knee and a line passing through the trochanter major and the center of rotation of the hip joint. The lateral and anterior offsets are the distances between the femoral axis and the center of rotation in the frontal and sagittal plane, respectively.

Apart from the influence of other surgical parameters, there are several mechanical parameters affecting the outcome of total hip arthroplasty (THA). In addition to leg length, a relevant aspect is the femoral offset, which should be restored as closely as possible to maximize long-term results^1^. The femoral offset describes the length of a line passing through the center of the femoral head and passing orthogonally through the axis of the femoral shaft. It can be observed from the frontal and sagittal planes and can be subdivided into a lateral and an anterior offset (Fig. 1). This reconstruction has a decisive influence on the periprosthetic hip muscle tension. In particular, improvements regarding the range of motion (ROM) and the lever arm of abductor muscles may be related to sufficiently reconstructed hip biomechanics and femoral offset^2,3^.

The focus of femoral offset on joint function after THA has been on the lateral femoral offset, since it is easily measured on standard anteroposterior radiographs. On the other hand, far less is known about the anterior femoral offset and its influence on joint function after THA.

As a function of stem type, there are different possibilities for the surgeon to take influence on the femoral offset. Conventional hip stems require resection of the femoral neck and, thus, allow different positions with regard to the lateral femoral offset via the choice of different implant geometries (e.g., CCD angle) and adjustments of the anterior femoral offset due to differently rotated positions within the femoral cavity^4^. The anterior femoral offset may be increased by rotating the stem in a more anterior way. However, this is done at the expense of a reduced lateral femoral offset, and the reconstruction of the physiological offset may become limited (Fig. 1).

On the other hand, calcar-guided short hip stems preserve the femoral neck basis and are adapted to the original anatomy of the femoral neck to physiologically reconstruct the anterior offset. Aligning the short hip stem in with an appropriate antetorsion angle allows for the independent reconstruction of the anterior offset. Since most short hip stems are not available with different CCD angles, the possibility of influence on the lateral femoral offset by adjustments due to the resection height of the femoral neck is limited^5^. The literature suggests a more anatomic reconstruction of the antetorsion angle with a calcar-guided short stem compared to a conventional stem, with the conventional stem introducing an malalignment of around 7°^6^.

To date, it is unknown how reconstruction differences in joint biomechanics due to conventional and calcar-guided short hip stems influence hip joint moments and gait parameters. Since these differences may affect muscle lever arms and joint mobility, the aim of this study was to determine whether differences in (a) four isokinetic hip joint moments (abduction, adduction, extension, flexion), (b) step length, (c) ROM, and (d) pelvic tilt during level walking between a conventional and a calcar-guided short hip stem reconstruction exist. The primary hypothesis is that the ratio of flexion to extension hip joint moment is increased due shortened lever arms of the hip extensors in case of treatment with a conventional stem compared to the calcar guided short stem.

## Materials and methods

### Subjects

**Figure 2:**
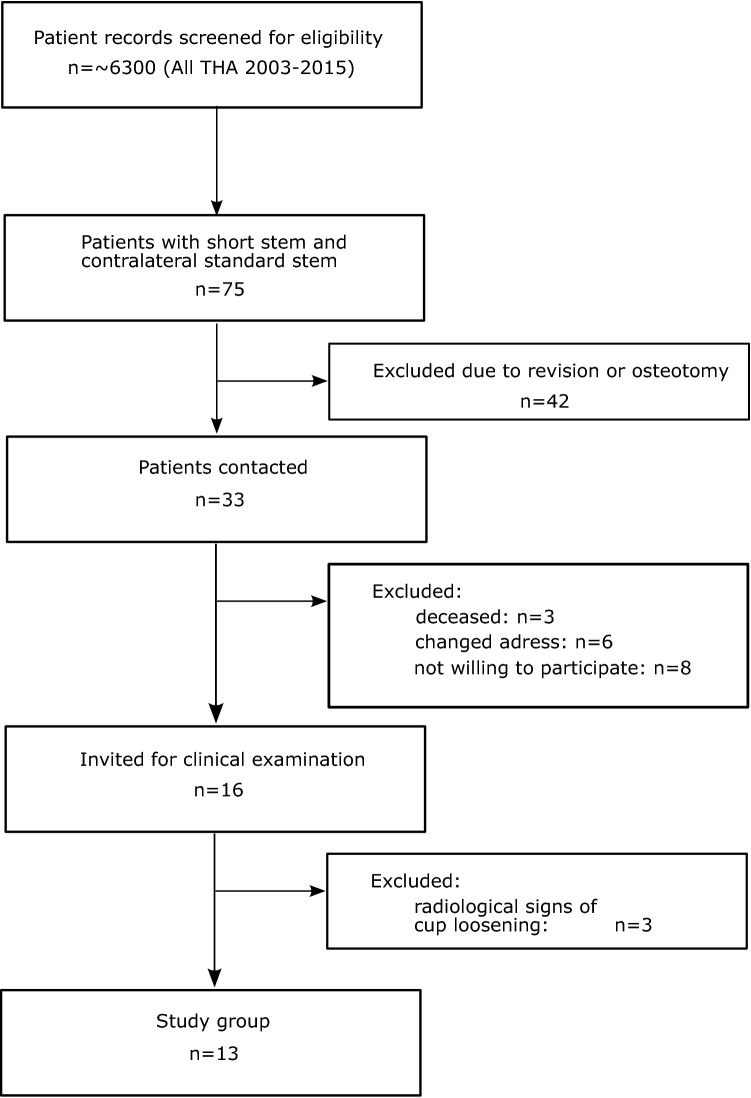
Flowchart of the patient selection process with reasons for exclusion.

**Figure 3:**
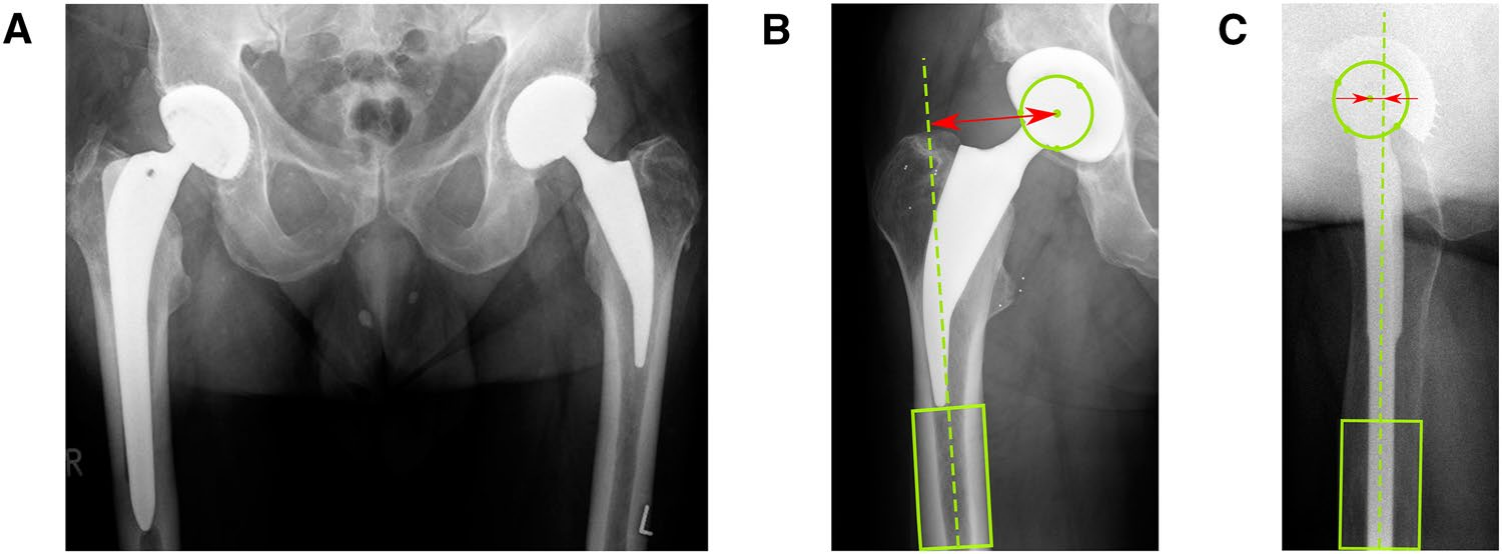
(**A**) Pelvic x-ray of a patient with a conventional hip stem (Bicontact) on the right side and a short hip stem (Metha) on the left side. (**B**) Measurement of the lateral offset on a clinical x-ray. (**C**) Measurement of the anterior offset in a Johansson positioning.

Within the records of our clinic, we retrospectively identified a cohort of 13 patients (Fig. 2) that had received both a conventional hip stem (Bicontact, Aesculap, Germany) and a contralateral, femoral neck-preserving, calcar-guided short hip stem (Metha, Aesculap, Germany) (Fig. 3A) between 2003 and 2015.

To assess the clinical outcome, the Harris Hip Score (HHS) was determined. Postoperative pelvic overview radiographs were taken during clinical routine follow-up examinations and the lateral femoral offset was surveyed. Lateral cross-table views were additionally analyzed to take the anterior femoral offset. The radiographs were standardized with the patella pointing strictly upward and were controlled by palpating both epicondyles to ensure parallel positioning of the knee with respect to the table. Radiographs were analyzed digitally using clinical image processing software (GEMED-PACS, GEMED mbH, Germany; Fig. 3).

To establish a comparison to healthy controls and determine the possible effects of side dominances, a further control cohort was included comprising 30 healthy adults, which were not matched to the patient cohort (Table 1).

**Table 1:** Demographic data of the patients and control group. Numeric data is given in mean and SD.

	Patients (n = 13)	Control (n = 30)
Age [years]	68.7 (6.1)	23.5 (3.2)
Sex	Female: n = 6, male: n = 7	Female: n = 15, male: n = 15
Mass [kg]	83.1 (14.4)	72.6 (12.1)
Height [m]	1.66 (0.49)	1.78 (0.09)
BMI [g/m^2^]	29.0 (6.0)	22.7 (2.1)
Leg dominance	Right: n = 13, Left: n = 0	Right: n = 26, Left: n = 4
Prosthesis side	Right: n = 6, Left: n = 7	Not applicable

### Measurements

**Figure 4:**
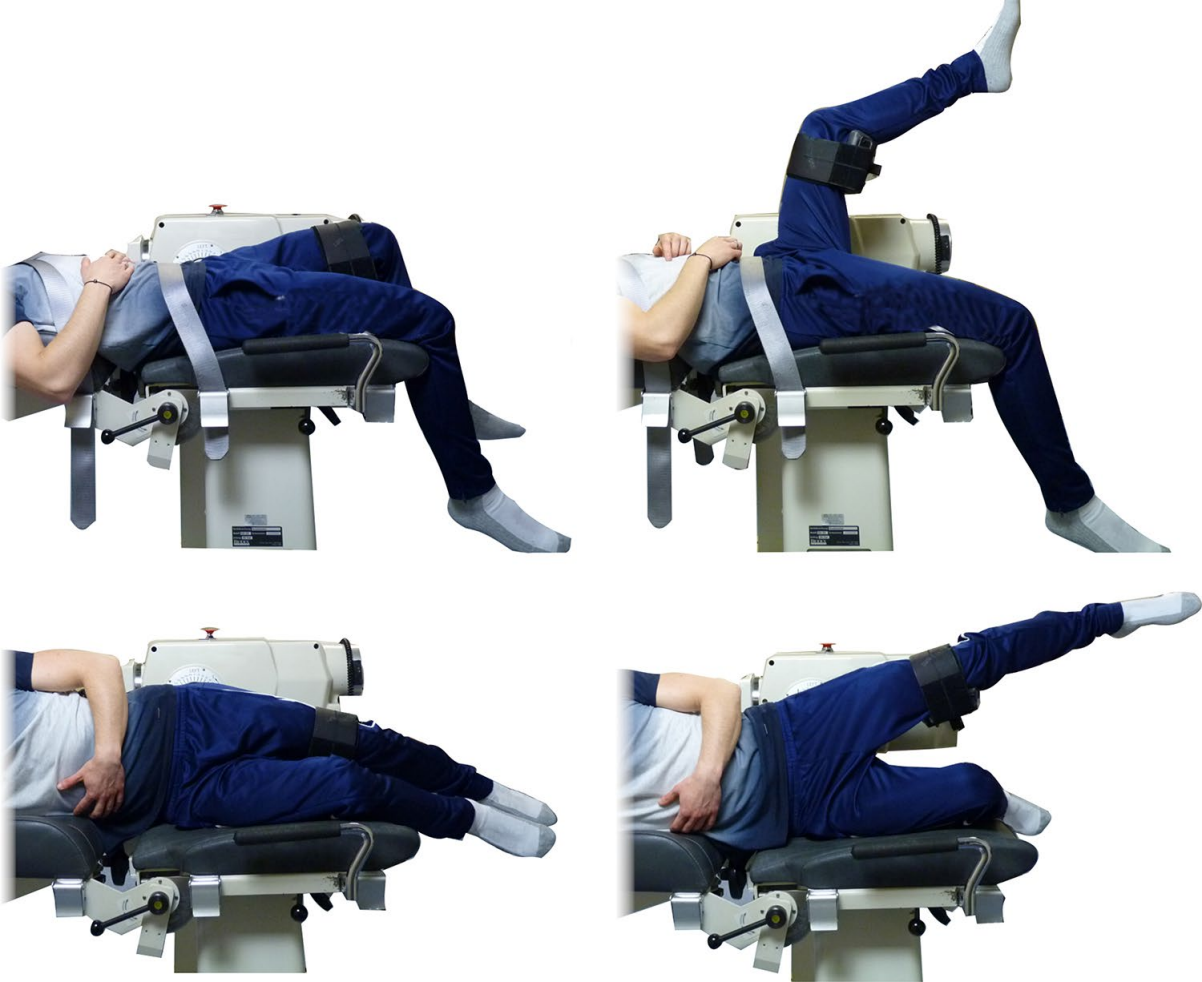
Positioning of subjects on the dynamometer and ROM limits for extension and flexion (top) as well as adduction and abduction measurement (bottom).

The dominant leg was determined by letting the patients and controls play with a ball using their preferred side^7^. In order to measure isokinetic hip moments, a set of tests (Fig. 4) from a previously published protocol was carried out on a dynamometer (Biodex System 2)^8^. Subjects were instructed to actively perform hip joint movements against the resistance of the device. For extension and flexion, the angular velocity was set to at 45°/s and the ROM from 10° to 65°^8^. For hip abduction and adduction, the velocity was set to 30°/s and the ROM from 0° to 35°^8^. Measurements for opposing directions (extension-flexion and abduction–adduction) were recorded in independent trials. After an initial warm-up and familiarization, each motion (extension, flexion, abduction, and adduction) was repeated 15 times with a break of 120 s every five repetitions (Fig. 4). Data was recorded at 200 Hz with customized software (LabView 2017, National Instruments, Austin, USA).

Gait parameters of patients were investigated using an infrared motion capturing system based on 12 MX-cameras at a sampling rate of 200 Hz (Nexus software, Version 1.8.5, Vicon Motion System Ltd., Oxford, UK). Leg length was measured with a measuring tape as the distance from the medial malleolus to the anterior superior iliac spine, via the knee joint. Retroreflective markers were placed to the body according to the Helen Hayes lower-body model^9^. Force plates measured ground reaction forces (Type BP400600, AMTI, Watertown, USA) with a sampling rate of 1 kHz. Each subject completed six trials during level walking with a self-selected velocity.

### Data processing and statistics

A low-pass filter (50 Hz cut-off) was applied to the dynamometer data to eliminate noise. The angle-dependent moment values were summarized by taking the integral of each trial and thus calculating work. Further processing was done on the mean work of all 15 trials. To account for the interindividual variability resulting from the physical capability of patients, the dynamometer data were summarized in two intraindividual indices:

(a) The symmetry index Isym:

Isym = (x_a_ − x_b_)/((x_a_ + x_b_)*0.5) with x representing work in one motion task and a/b being right/left for reference subjects and conventional/short hip stem for the patients.

(b) The directional index Idir:

Idir = (y_a_ − y_b_)/((y_a_ + y_b_)*0.5) with a/b representing the work in adduction/abduction and extension/flexion.

Leg dominance is not accounted for in any of the indices.

Considered gait parameters were step length, ROM (extension/flexion, abduction/adduction, pelvic tilt, pelvic obliquity, pelvic rotation), and peak moments (extension/flexion, abduction/adduction). Additionally, the time integrals were calculated for the first 30% of the gait cycle to investigate the effect of the implant type on the adductor muscles^10^.

Analysis was conducted in R^11^ and figures were produced using the package ggplot2^12^. Statistical comparison utilized unpaired Mann–Whitney-U-tests for the Isym and paired Wilcoxon signed-rank tests for Idir, gait, and radiological parameters. Results with p < 0.05 were regarded as statistically significant.

### Ethics approval

All procedures described in this paper were carried out in accordance with relevant guidelines and regulations, approved by the local ethics committee (Ethics Committee of Hannover Medical School, No. 7559 and 7561), and registered in the German Clinical Trial Registry (DRKS00012654). All subjects and patients provided informed written consent.

## Results

Among the recruited patients (n = 13), nine were able to perform all considered isokinetic measurements, and twelve were able to complete gait analysis. Thus, four patients refused the abduction tests due to motor function impairments and one patient refused the gait analysis. A pelvic overview x-ray was available for all patients, whereas a lateral cross-table view including both hip joints was available for eight patients. All the 30 control subjects successfully performed the isokinetic measurements.

### Demographic, clinical and radiological parameters

All patients were right leg-dominant (Table 1). The average HHS was 80.3 points (range, 59 to 91 points). The leg with the short stem prosthesis was 2.1 ± 10.5 mm longer than the leg with the conventional prosthesis (p = 0.255). The anterior femoral offset was significantly higher (p = 0.014) in legs treated with the short hip stem (25.9 ± 7.8 mm) compared to legs treated with the conventional hip stem (15.1 ± 9.3 mm). The lateral femoral offset did not differ significantly (p = 0.162) between legs with the short hip stem (46.7 ± 6.5 mm) and legs with the conventional hip stem (42.9 ± 4.9 mm).

In the control cohort 26, participants were right leg-dominant (Table 1).

### Hip moments

**Figure 5:**
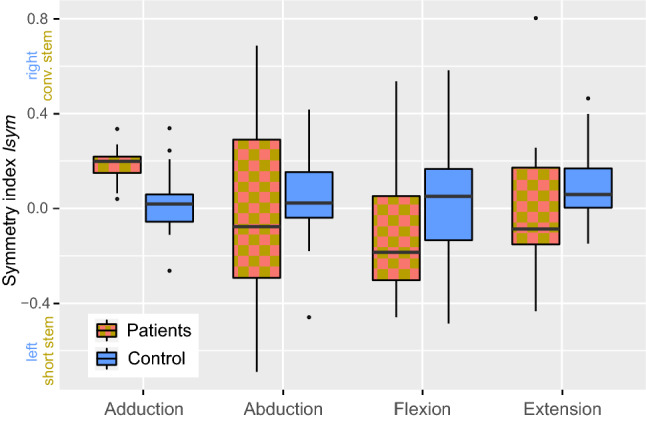
Symmetry index Isym for leg side comparisons regarding hip moment directions. An index above 0 indicates a stronger moment on the side with the conventional stem in patients and on the right side with controls, respectively.

Except for adduction moment (p < 0.001) in legs treated with the conventional hip stem, the mean symmetry index Isym showed balanced side dominances (Fig. 5). Regarding the control subjects, no side dominances were shown with mean Isym values for adduction (0.02 ± 0.11), abduction (0.05 ± 0.17), extension (0.09 ± 0.15) or flexion (0.04 ± 0.24).

**Figure 6:**
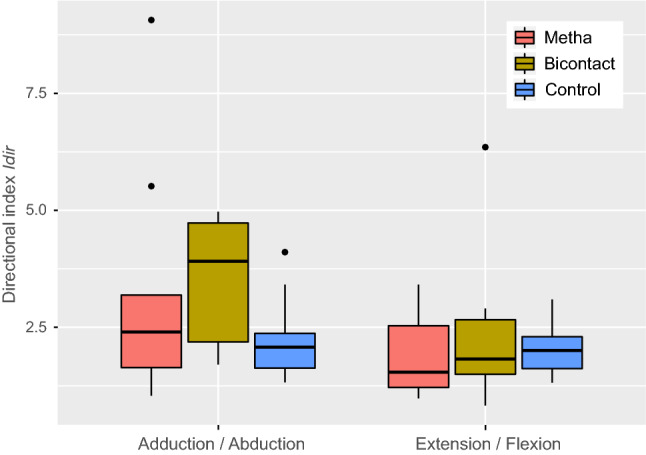
Directional index Idir of antagonistic hip moments: (**a**) adduction/abduction, (**b**) extension/flexion. Ratios on the short stem side are colored in red, the conventional stem side in yellow, and the control group in blue. Positive values indicate hip adduction and extension moment dominance.

Ratios of antagonistic hip moments as described by the directional index Idir were comparable between patients and controls, particularly regarding the extension/flexion ratio (Fig. 6). In the control group, the mean Idir for both sides in adduction/abduction was 2.13 ± 0.64 and that for extension/flexion was 2.00 ± 0.47. The mean Idir for adduction/abduction was 3.26 ± 2.53 and that for extension/flexion was 1.81 ± 0.76 for legs treated with the short hip stem, whereas these values were 3.49 ± 1.37 and 2.17 ± 1.41, respectively, for legs treated with the conventional hip stem. The Idir of legs treated with the conventional hip stem showed a significantly stronger dominance of the hip adduction moment compared to the control group (p = 0.008). In contrast, there were no significant differences in Idir between legs treated with the short hip stem and the control group (p = 0.25) and between both hip stems (p = 0.55).

### Gait analysis

The step length was significantly increased (p = 0.03) for legs treated with the short hip stem (0.63 ± 0.06 m) compared to that in legs treated with the conventional hip stem (0.61 ± 0.06 m), while all further parameters did not differ (Table 2).

**Table 2:** Mean and SD of gait analysis parameters.

	Hip stem		p-value
	Short	Conventional	
Step length [m]	0.63 (0.06)	0.61 (0.06)	0.03
ROM extension/flexion [°]	39.8 (5.3)	40.1 (4.9)	1.00
ROM abduction/adduction [°]	9.70 (2.13)	9.66 (2.53)	1.00
ROM pelvic tilt [°]	2.63 (1.39)	2.64 (1.51)	0.88
ROM pelvic obliquity [°]	5.47 (2.76)	5.47 (2.61)	0.88
ROM pelvic rotation [°]	10.3 (5.1)	10.2 (5.3)	0.35
Peak moment extension/flexion [Nm/kg]	1.19 (0.38)	1.10 (0.39)	0.48
Peak moment abduction/adduction [Nm/kg]	0.97 (0.26)	0.92 (0.31)	1.00
Time integral 0% to 30% gait cycle moment extension/flexion [Nm/kg*0.01]	0.03 (0.13)	0.02 (0.06)	0.85
Time integral 0% to 30% gait cycle moment abduction/adduction [Nm/kg*0.01]	0.20 (0.04)	0.18 (0.06)	0.63

## Discussion

The aim of the present study was to determine differences in joint function after short stem THA using a calcar-guided implant compared to conventional THA, under the assumption that retaining the femoral neck may provide benefits regarding the proper reconstruction of joint mechanics^13^. The first aspect analyzed was whether there is an actual difference in biomechanical parameters between the two types of stem implants. However, the primary hyposthesis of an increase in ratio of flexion to extension hip joint moment could not be confirmed. The radiological evaluation of the lateral and anterior femoral offset confirmed that there was indeed a significant difference: the short stem produces a significantly higher anterior femoral offset than the conventional stem, whereas the lateral femoral offset was comparable between stems. Since radiological data allowing the measurement of the preoperative anterior offset were not available, it can only be stated that there is a significant difference between the implants, but not which implant achieves more physiological restoration of joint mechanics. However, data from other studies suggest that the short stem leads to a more anatomical reconstruction^6,13^.

No relevant bias arising from side-dominance was observed since the absolute Isym values were below 0.1. The low difference between sides corresponds to the results of other studies evaluating side dominance, which report a maximum difference of 0.8–13% between dominant and non-dominant legs^14–16^. Furthermore, all patients had a dominant right leg; therefore, a possible effect should be canceled out by the even distribution of the short and conventional stem on both sides.

Analysis revealed that most of the parameters assessed by gait analysis did not show a significant difference between the two sides, which agrees well with other data analyzing gait analysis parameters after short stem arthroplasty compared to conventional stem arthroplasty^17,18^. The observed longer step length of the short-stem side may be attributed to changes in joint mechanics of either the swinging leg side or the standing leg side. Whilst the leg with the short stem is 0.002 m longer on average, this does not explain the significant difference in step length. Regarding the other parameters from gait analysis, whether the method of gait analysis itself is sensitive enough to detect the effect of minor changes in muscle lever arms remains open to future discussion. In our study, the use of a dynamometer seemed to be a more promising approach because it eliminates biases, such as compensatory strategies during gait.

Analysis of the results of the isokinetic measurement of hip moments showed more specific differences. While extension, flexion, and abduction moments were not significantly different between stems, the adduction moment was significantly higher on the side of the conventional stem (p < 0.001). To reduce the variation caused by the individual physiological condition of each patient, the ratios of opposing moments were analyzed, allowing for a comparison to the group of healthy participants.

The ratio between abduction and adduction was significantly different between the conventional stem group and the control group (p = 0.008), but it was not significant between the short stem group and the control group (p = 0.25). This finding that implantation of the conventional stem caused changes in joint kinematics that did not occur with the short stem implies that short stem THA may lead to a more physiologic joint function than that with conventional stem THA. This change in the abduction and adduction moments could be explained by the three-dimensional change of the direction of muscle fibers when the anterior offset is not reconstructed anatomically. While the abduction/adduction lever arm is predominantly defined by the lateral offset, the anterior offset changes the muscle paths three-dimensionally in a non-neglectable way affect the abduction and adduction moments.

To our knowledge, this is the first study to compare different implant types interindividually by means of isokinetic measurements of moments and a gait analysis. Other studies have focused on differences in hip moments either between the pre- and postoperative condition or between an operated and a healthy limb, but could not detect significant differences^19^.

Nonetheless, this study has several limitations. First, the case number was small, and the scatter of isokinetic data of the THA patients was rather high. Second, the surgical approach to the hip joint had not been considered. Minimal invasiveness has been shown to be improve rehabilitation, gait, and joint function in the short term^20,21^. However, the results of many studies suggest that this beneficial effect may only be present within the first months after surgery^22–24^. Since the time between surgery and examination was at least 4 years for all the patients, it can be assumed that the influence of the surgical approach on the presented results is negligible.

Furthermore, this study focused on objective parameters regarding joint function, whereas the patients’ subjective perception of differences remained disregarded. Although the assessed HHS includes subjective parameters, it does not differentiate between the two different sides.

Third, the control group was not age-matched to the patient group. While the control group showed higher absolute values in hip moments, this effect should be accounted for by the usage of the symmetry index Idir.

In conclusion, the results of this study indicate that implantation of a calcar-guided short stem leads to a difference in the reconstruction of the radiologically measured anterior femoral offset compared to that with a conventional stem. The joint function measured by isokinetic measurements (specifically the directional index) remains comparable to controls after short stem arthroplasty, but shows slight changes after conventional stem arthroplasty.

## Data Availability

The datasets generated during and/or analysed during the current study are available from the corresponding author on reasonable request.
